# Information Seeking Behaviors and Preferences of Cancer Survivors: A Cross-sectional Study in Shanghai, China

**DOI:** 10.1007/s13187-025-02628-9

**Published:** 2025-05-26

**Authors:** Mengying Liu, Yuan Xu, Jie Song, Xiaojing Hu, Chunlin Jin, Ziping Liu, Ruijia Li, Minxing Chen

**Affiliations:** 1 College of Pharmacy, Anhui Xinhua University, Hefei, 230088 China; 2 Shanghai Health Development Research Center, Shanghai Medical Information Center, Shanghai, China

**Keywords:** Cancer survivors, Information seeking behaviors, Information seeking preferences

## Abstract

The information needs of cancer survivors are numerous and chronically difficult to meet, and the information sources currently available to them are far from ideal. The purpose of this study was to investigate the information-seeking behaviors and preferences of cancer survivors and explore the differences in information needs among cancer survivors of different types in Shanghai, China. A quota sampling method was used to select cancer survivors living in all districts of Shanghai as the study population. Data collection used the Unmet Information Needs Scale for Cancer Patients. ANOVA were used to assess differences in unmet need scores among subgroups. The mean age of 4195 cancer survivors was 63.2 ± 7.4 years, comprising 823 males (19.6%) and 3372 females (80.4%). Among the current sources of information, 36.2% of cancer survivors opted for consultations with tertiary-level doctors. In terms of desired sources, 43.9% preferred tertiary-level doctors. Regardless of the current or future situation, tertiary-level doctors, patient friends, and primary care doctor remain the top three preferred sources for health information. Kidney cancer, metastatic cancer, and nasopharyngeal cancer demonstrated the highest information requirement scores, scoring 39.4, 37.5, and 36.6, respectively. Cancer survivors of different cancer types showed differences in focus and information needs. Current and expected information sources for cancer survivors differ at different stages of survival. Tertiary-level doctors are the most popular providers of information. Unmet information needs for different cancer types are diverse and complex.

## Introduction

Cancer constitutes a significant public health concern worldwide. Predictions indicate a concerning rise in cancer incidence, with new cases expected to soar by 77% by 2050, surpassing 35 million, compared to 20 million in 2022 [1]. This surge is anticipated predominantly in countries with lower to medium Human Development Indices. China, in particular, has witnessed a steady uptrend in both incidence and mortality rates of malignant tumors since 2000, confronting an escalating cancer burden [2–4]. The landscape of cancer research is marked by significant breakthroughs, introducing novel and more effective treatment modalities each year. Technological progressions such as early detection methods, precision medicine, and immunotherapeutic approaches have significantly enhanced survival outcomes, contributing to improve the quality of life of cancer survivors [5].

A cancer survivor, as defined, is an individual who has completed treatment aimed at curing the disease and is either disease-free or in remission [6]. However, the implications of surviving cancer transcend the physical consequences of the illness and its therapies in an academic context. It involves managing a range of immediate and prolonged consequences, such as unfavorable responses, the potential for subsequent malignancies, and various psychosocial difficulties [7, 8]. It has been observed that navigating life post-treatment often presents more challenges for survivors than the treatment phase itself, indicating a critical need for a holistic, patient-centered approach in healthcare. This approach underscores the significance of collaborative decision-making, patient self-management, empowerment, and the resolution of their unmet informational requirements [9].

Information assumes a critical role in enabling cancer survivor to manage the challenges to their quality of life arising from the diagnosis and treatment of cancer [10, 11]. Access to relevant information is crucial as it can mitigate uncertainties, enhance a sense of personal control, assist in making informed decisions regarding treatment options, and increase satisfaction with treatment outcomes [12, 13]. However, cancer survivors are confused when seeking information about their diagnosis, potential coping strategies, prognosis, treatment alternatives, and supportive care measures [14]. The wealth of information from doctors, caregivers, family members, patient friends, and websites is varied but of uncertain quality, often making it difficult for cancer survivors to access truly beneficial information. In fact, cancer survivors’ need for effective information is very high and unmet for a long time.

Meanwhile, the preferences for and priorities of information vary among cancer survivors, and their information-seeking behaviors can change over time [14, 15]. Various sources, including healthcare professionals, the internet, family, friends, and peer support groups, have been identified as primary information channels for cancer survivors [16, 17]. Despite the diversity of available sources, there exists a notable research gap concerning the information needs of cancer survivors at different stages of their survivorship journey, particularly in relation to varying preferences for information sources. Additionally, studies aimed at understanding the comprehensive informational needs of cancer survivors across different types in China have been limited by small sample sizes, thus failing to reflect the broader spectrum of needs accurately [18–20].

Shanghai, China’s leading economic hub, has a total population of approximately 24.85 million [21]. According to the latest surveillance data, in 2020, Shanghai reported 85,000 new cancer cases, with an incidence rate of 576 per 100,000 population [22]. The top three cancers were lung cancer, colorectal cancer, and thyroid cancer. The study employs a comprehensive questionnaire to investigate the present status and areas lacking in cancer-related information acquisition among cancer survivors in Shanghai. The study aims to elucidate disparities in information retrieval methods among cancer survivors at different stages of survivorship and to pinpoint specific informational requirements according to cancer type. As a basic appeal, information needs are met that can effectively help cancer survivors manage their health and well-being after diagnosis. In addition, cancer survivors deserve more attention for such a vulnerable group, and patient-centered cancer survivor care programs need to be continuously improved.

## Material and Methods

### Study Design and Population

This research was carried out in Shanghai, China. Quota sampling was conducted in all areas of Shanghai (16 districts), and 300 questionnaires were distributed by trained research assistants in each district. After excluding invalid questionnaires, 4195 questionnaires were included in the final statistical analysis, with a valid response rate of 99.4%. The sampling period spanned from April 15 to April 21, 2022, ensuring a comprehensive representation of the cancer survivor population within the city. Since 1995, the China Anti-Cancer Association has designated April 15–21 each year as the National Cancer Prevention and Treatment Publicity Week [23]. The campaign calls for community-wide attention to the health management of cancer patients and aims to achieve “integrated medicine” from the resources of the medical profession and new technological tools. We surveyed the needs of cancer patients in Shanghai during the 28 th National Cancer Awareness Week in 2022 using the online questionnaire. Eligibility criteria included participants aged 18 years or older, in a stable or recovery phase of cancer, residing in Shanghai for at least 3 months prior to the study, and capable of providing informed consent and comprehending the survey questions. To ensure data reliability, we implemented strict exclusion criteria: (a) exclusion of acute-phase patients: Patients undergoing active treatment (e.g., chemotherapy, radiotherapy) or hospitalized for acute symptoms were excluded, as their condition might impair their ability to complete the survey reliably. (b) Cognitive screening: Participants with documented cognitive impairments (e.g., dementia) or those unable to provide informed consent were excluded. Cognitive status was assessed via medical records or a brief screening tool during recruitment. (c) Proxy respondents: For survivors with temporary physical limitations, trained interviewers administered the survey in person or via phone to minimize proxy bias. Relatives or caregivers were only permitted to assist with logistical aspects (e.g., reading questions aloud) but not to interpret or answer on behalf of the participant. (d) Validation checks: A subset of responses was cross-verified with medical records or follow-up interviews to ensure consistency. These measures ensured that the data primarily reflected the firsthand experiences of cognitively intact survivors, reducing potential bias from proxy respondents.

### Data Collection

The data collection instruments included a General Information Questionnaire and an Unmet Information Needs Scale for Cancer Patients, both developed by the research team to align with the study’s objectives. The questionnaire for this study was referenced from the Supportive Care Needs Survey-Short Form (SCNS-SF34) [24] and the Short Form for Unmet Needs of Cancer Patients (SF-SUNS) [25]. And we simplified and adapted the questionnaire due to language and cultural differences between countries that may affect the measurement of patient-reported outcomes [26, 27]. We conducted an in-depth interview with cancer patients and modified the questionnaire according to the high-frequency words in patient information demand in the interview. We conducted Delphi expert consultations to revise our questionnaire in December 2021, January 2022, and March 2022. Experts suggested that we should include cancer survivors’ needs for disease burden and commercial health insurance in the questionnaire scale. Also, it should be ensured that all the contents of the questionnaire are easy to understand for participants with different levels of education. In addition, a pre-survey including 60 participants was conducted to ensure that each question in the questionnaire scale was set to match the Chinese population.

The General Information Questionnaire gathered demographic and clinical characteristics, including gender, age, cancer stage, marital status, education level, employment status, income, type of medical insurance, and physical activity levels. The Unmet Information Needs Scale was structured around four dimensions: prevention, treatment, rehabilitation, and lifestyle information, with items rated on a 5-point Likert scale ranging from “not needed” to “very much needed.” The total Cronbach’s alpha coefficient of the scale was 0.87, and the coefficients of all dimensions were greater than 0.80. Validity analysis showed that the Kaiser–Meyer–Olkin value was 0.98 (*P* < 0.05). This scale enabled the assessment of patients’ informational needs across various aspects of cancer care and survivorship [28]. Additionally, we explored patients’ preferences for sources of health management advice, asking about their current and ideal sources of information. This aspect aimed to identify gaps between available and preferred information resources among cancer survivors.

Data were collected via an online platform, where surveys were disseminated across various districts to ensure representation across geographic and demographic dimensions. Quality control measures, such as IP filtering and time limit restrictions, were implemented to ensure the integrity of the survey responses. Survey facilitators and research team members conducted a thorough review of the completed questionnaires for accuracy and completeness.

### Statistical Analysis

The gathered data underwent meticulous validation procedures, including double-entry error checking, prior to statistical analysis using the SPSS software (version 26.0). We performed descriptive analyses to summarize demographic characteristics and needs scores across different dimensions. Mean scores ± standard deviations were computed for continuous variables, while frequencies and percentages were determined for categorical variables. ANOVA were conducted to examine the effects of demographic characteristics on unmet needs scores, identifying significant differences in information needs among different patient groups. Through this methodological approach, we aimed to comprehensively understand the informational needs and preferences of cancer survivors in Shanghai, providing valuable insights into how best to support them through targeted information delivery and support services.

## Results

Among the 4195 cancer survivor surveyed, 823 were male (19.6%) and 3372 were female (80.4%), with an average age of 63.23 ± 7.4 years (Table 1). The majority of cancer survivor were retired (87.1%), and a significant portion had a monthly income below 6000 RMB (79.9%). Localized cancer was present in 92.9% of cancer survivor, while metastatic cancer was present in 7.1%.

**Table 1:** Baseline characteristic of the study participants

Characteristic	Male (*N* = 823)		Female (*N* = 3372)		Total (*N* = 4195)	
	*N*	%	*N*	%	*N*	%
Age (mean ± SD) (years)	65.93 ± 7.32		62.57 ± 7.30		63.23 ± 7.43	
18–65	379	46.1	2159	64	2538	60.5
≥ 65	444	53.9	1213	36	1657	39.5
Marital status						
Single/widowed	73	8.9	486	14.4	559	13.3
Married	750	91.1	2886	85.6	3636	86.7
Education level						
≤ 9 years	341	41.4	1592	47.2	1933	46.1
9–12 years	302	36.7	1343	39.8	1645	39.2
≥ 12 years	180	21.9	437	13	617	14.7
Working status						
Employed	96	11.7	446	13.2	542	12.9
Unemployed/retired	727	88.3	2926	86.8	3653	87.1
Physical activity						
Extremely active	254	30.9	581	17.2	835	19.9
Vigorously active	147	17.9	597	17.7	744	17.7
Moderately active	336	40.8	1415	42.0	1751	41.7
Sedentary	86	10.4	779	23.1	865	20.6
Average monthly income (RMB)						
≤ 3000	187	22.7	790	23.4	977	23.3
3001–6000	450	54.7	1926	57.1	2376	56.6
6001–9000	129	15.7	448	13.3	577	13.8
≥ 9000	57	6.9	208	6.2	265	6.3
Medical insurance						
Basic medical insurance	272	33	1195	35.4	1467	35
Employee medical insurance	526	63.9	2010	59.6	2536	60.5
Commercial medical insurance	20	2.4	161	4.8	181	4.3
None	5	0.6	6	0.2	11	0.3
Time since diagnosis (years)						
≤ 2	85	10.3	242	7.2	327	7.8
2–5	174	21.1	683	20.3	857	20.4
5–10	307	37.3	1219	36.2	1526	36.4
≥ 10	257	31.2	1228	36.4	1485	35.4
Disease staging						
Stage I	227	27.6	935	27.7	1162	27.7
Stage II	201	24.4	1054	31.3	1255	29.9
Stage III	169	20.5	583	17.3	752	17.9
Stage IV	52	6.3	119	3.5	171	4.1
Not sure	174	21.1	681	20.2	855	20.4

**Fig. 1:**
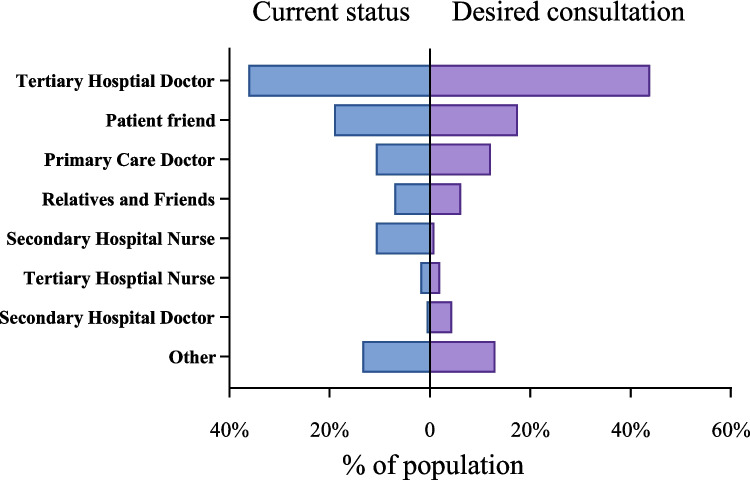
The current and desired sources of information consultation

Figure 1 illustrates the current and desired sources of information consultation. Among the current sources of information, 36.2% of cancer survivor chose tertiary-level doctors, 19.1% opted for patient friends (support groups), and 10.8% selected both primary care doctors and secondary hospital nurses. In terms of desired sources, 43.9% expressed a preference for tertiary-level doctors, 17.5% favored patient friends, and 12.1% indicated a preference for primary care doctors. Regardless of the current or future situation, tertiary-level doctors, patient friends, primary care doctors, and secondary hospital nurses remain the primary sources for health information.

Descriptive statistics on the health information sources of cancer survivors by diagnosis time are presented in Table 2. Tertiary-level doctors are consistently identified as the primary preference for health information among cancer survivors, both presently and prospectively. There were statistically significant differences in the information sources among cancer survivors at different stages of survival, specifically regarding tertiary hospital nurses and patient friends (*P* < 0.05). In terms of desired sources of health information, there were significant differences in preferences for secondary hospital nurses among cancer survivors at different survival stages (*P* < 0.05).

**Table 2:** Status and expectations of sources of information and counseling for cancer survivors stratified by different stages of survival

Time since diagnosis (years)		Tertiary hospital doctor		Tertiary hospital nurse		Secondary hospital doctor		Secondary hospital nurse		Primary care doctor		Relatives and friends		Patient friend		Other	
		*N* (%)	*P*	*N* (%)	*P*	*N* (%)	*P*	*N* (%)	*P*	*N* (%)	*P*	*N* (%)	*P*	*N* (%)	*P*	*N* (%)	*P*
Current status	< 2	180 (8.1)	0.685	4 (3.4)	**0.022**	13 (6.0)	0.628	3 (7.3)	0.786	54 (8.2)	0.831	36 (8.4)	0.796	104 (8.9)	**0.017**	57 (6.9)	0.308
	2–5	443 (20.0)		36 (30.5)		41 (19.0)		11 (26.8)		129 (19.5)		81 (18.8)		218 (18.7)		178 (21.6)	
	5–10	814 (36.8)		40 (33.9)		79 (36.6)		14 (34.1)		248 (37.6)		156 (36.2)		400 (34.3)		283 (34.4)	
	> 10	775 (35.0)		38 (32.2)		83 (38.4)		13 (31.7)		229 (34.7)		158 (36.7)		445 (38.1)		305 (37.1)	
Desired consultation	< 2	204 (8.5)	0.229	8 (7.3)	0.351	13 (5.4)	0.156	1 (2.0)	**0.017**	59 (9.0)	0.255	34 (10.1)	0.408	76 (8.0)	0.323	44 (6.2)	0.291
	2–5	489 (20.5)		30 (27.3)		40 (16.6)		16 (32.0)		120 (18.3)		67 (19.9)		174 (18.3)		150 (21.3)	
	5–10	857 (35.8)		36 (32.7)		93 (38.6)		23 (46.0)		251 (38.2)		116 (34.4)		352 (37.0)		250 (35.5)	
	> 10	841 (35.2)		36 (32.7)		95 (39.4)		10 (20.0)		227 (34.6)		120 (35.6)		349 (36.7)		261 (37.0)	

ANOVA were conducted to examine the effects of status and expectations of sources of information among different time since diagnosis (< 2 vs. 2–5 vs. 5–10 vs. > 10 years) groups. *P* < 0.05 indicated that the difference among the groups was statistically significant

Table 3 details the information need scores among survivors of different cancer types. A significant majority, 3896 (92.9%) of the patients, had primary cancers, significantly higher than those with metastatic cancers, 299 (7.1%). Among primary cancers, the most common were breast cancer (39.1%), colorectal cancer (10.3%), and tracheal, bronchial, and lung cancers (10.2%). Kidney cancer, metastatic cancer, and nasopharyngeal cancer had the highest information need scores, with scores of 39.4, 37.5, and 36.6, respectively. Different cancer survivors showed variance in their focus and need for information. Among breast cancer survivors, there was a higher focus and need for information on carcinogenic factors and medical insurance, with scores of 3.33 and 2.97, respectively. Colorectal cancer survivors exhibited a greater interest in information regarding carcinogenic factors, medical insurance, and survival rates, with scores of 3.25, 3.04, and 2.94, respectively. Tracheal, bronchial, and lung cancer survivors prioritized information on carcinogenic factors the most (score 3.30), followed by medical insurance information (score 3.11), and then survival rate, scoring 3.03. Metastatic cancer survivors had higher needs for information on carcinogenic factors, medical insurance, and survival rate, with scores of 3.75, 3.56, and 3.44, respectively.

**Table 3:** Information needs scores of cancer survivors from different types of cancer

Cancer type	*N* (%)	Total need score	Prevention information need (mean (SD))			Treatment information need (Mean (SD))			Rehabilitation information need (Mean (SD))			Living information need (Mean (SD))		
			Genetic	Carcinogenic factors	Recurrence monitoring	Oncologists	Survival rate	Complementary therapies	Continuing care	Symptom management	Psychological counselling	Medical Insurance	Financial assistance	Sexuality
Primary cancer	3896 (92.9)	31.9 (14.3)	2.69 (1.59)	3.36 (1.49)	2.64 (1.50)	2.88 (1.57)	2.91 (1.50)	2.60 (1.52)	2.61 (1.49)	2.51 (1.49)	2.15 (1.40)	3.03 (1.52)	2.86 (1.56)	1.69 (1.25)
Breast cancer	1639 (39.1)	31.4 (14.1)	2.69 (1.59)	3.33 (1.50)	2.60 (1.50)	2.83 (1.55)	2.83 (1.48)	2.53 (1.49)	2.57 (1.47)	2.47 (2.40)	2.10 (1.35)	2.97 (1.50)	2.82 (1.55)	1.64 (1.21)
Colorectal cancer	430 (10.3)	32.5 (13.8)	2.74 (1.56)	3.25 (1.48)	2.61 (1.45)	2.91 (1.54)	2.94 (1.49)	2.68 (1.53)	2.71 (1.52)	2.53 (2.39)	2.23 (1.46)	3.04 (1.54)	2.86 (1.55)	1.87 (1.39)
Tracheal, bronchial, and lung cancers	428 (10.2)	32.4 (14.9)	2.61 (1.56)	3.30 (1.51)	2.73 (1.47)	2.93 (1.55)	3.03 (1.49)	2.75 (1.51)	2.62 (1.48)	2.57 (2.43)	2.21 (1.40)	3.11 (1.48)	2.93 (1.57)	1.69 (1.22)
Stomach cancer	290 (6.9)	32.5 (15.1)	2.77 (1.62)	3.44 (1.46)	2.76 (1.55)	2.87 (1.60)	2.99 (1.52)	2.71 (1.58)	2.62 (1.55)	2.55 (2.37)	2.31 (1.46)	3.09 (1.54)	2.91 (1.60)	1.86 (1.34)
Thyroid cancer	242 (5.8)	32.9 (14.9)	2.51 (1.58)	3.28 (1.57)	2.44 (1.52)	2.73 (1.57)	2.85 (1.55)	2.59 (1.53)	2.45 (1.52)	2.35 (2.16)	2.17 (1.45)	2.88 (1.55)	2.77 (1.62)	1.74 (1.29)
Cervical cancer	163 (3.9)	34.3 (15.5)	2.82 (1.75)	3.44 (1.61)	2.85 (1.61)	2.80 (1.65)	2.94 (1.62)	2.79 (1.64)	2.83 (1.64)	2.72 (2.46)	2.25 (1.55)	3.25 (1.61)	3.03 (1.72)	1.93 (1.50)
Ovarian cancer	109 (2.6)	33.7 (16.6)	2.77 (1.65)	3.47 (1.53)	2.63 (1.50)	2.95 (1.61)	3.06 (1.58)	2.60 (1.53)	2.57 (1.46)	2.61 (2.32)	2.25 (1.44)	3.11 (1.50)	2.90 (1.47)	1.52 (1.09)
Liver cancer	83 (2.0)	30.4 (14.2)	2.59 (1.67)	3.36 (1.56)	2.93 (1.62)	3.18 (1.68)	3.14 (1.56)	2.95 (1.64)	2.87 (1.67)	2.67 (2.34)	2.49 (1.53)	3.20 (1.55)	3.11 (1.59)	1.84 (1.35)
Prostate cancer	37 (0.9)	30.8 (14.8)	1.95 (1.51)	3.14 (1.60)	2.76 (1.61)	3.30 (1.71)	2.89 (1.52)	2.78 (1.72)	2.70 (1.60)	2.78 (2.27)	2.22 (1.40)	3.16 (1.56)	3.03 (1.46)	1.78 (1.40)
Bladder cancer	37 (0.9)	25.1 (14.1)	2.16 (1.52)	2.62 (1.66)	1.97 (1.36)	2.35 (1.62)	2.08 (1.42)	2.22 (1.60)	1.97 (1.38)	1.81 (1.35)	1.65 (1.09)	2.51 (1.73)	2.46 (1.59)	1.27 (0.77)
Kidney cancer	31 (0.7)	39.4 (13.4)	2.81 (1.42)	3.68 (1.30)	2.55 (1.48)	2.58 (1.61)	2.94 (1.39)	2.52 (1.53)	2.52 (1.46)	2.52 (2.02)	2.16 (1.34)	2.90 (1.58)	2.94 (1.46)	1.45 (1.06)
Endometrial cancer	28 (0.7)	29.3 (14.8)	2.07 (1.22)	2.61 (1.52)	2.00 (1.22)	2.14 (1.35)	2.39 (1.50)	2.18 (1.44)	2.14 (1.38)	1.82 (1.39)	1.50 (0.69)	2.43 (1.45)	2.07 (1.22)	1.21 (0.83)
Lymphoma	23 (0.5)	32.4 (13.5)	2.74 (1.71)	3.57 (1.41)	2.87 (1.60)	3.04 (1.67)	3.22 (1.45)	2.91 (1.73)	2.61 (1.62)	2.57 (1.90)	2.09 (1.56)	3.39 (1.50)	3.30 (1.55)	2.00 (1.54)
Nasopharyngeal cancer	22 (0.5)	36.6 (15.3)	2.45 (1.68)	3.00 (1.60)	2.45 (1.60)	2.86 (1.67)	3.00 (1.57)	2.41 (1.53)	2.45 (1.57)	2.00 (1.45)	1.91 (1.27)	2.82 (1.74)	2.55 (1.57)	1.36 (0.90)
Esophageal cancer	20 (0.5)	34.3 (14.4)	2.50 (1.57)	3.05 (1.36)	2.50 (1.47)	2.75 (1.52)	2.50 (1.50)	2.65 (1.57)	2.85 (1.57)	2.60 (1.90)	1.95 (1.40)	2.75 (1.62)	2.45 (1.50)	1.85 (1.50)
Pancreatic cancer	14 (0.3)	31.5 (12.8)	2.93 (1.64)	4.29 (1.20)	3.29 (1.33)	3.86 (1.46)	3.93 (1.39)	3.57 (1.51)	2.93 (1.49)	3.07 (2.21)	2.50 (1.51)	3.64 (1.65)	3.93 (1.39)	1.43 (1.16)
Gallbladder/ductal cancer	10 (0.2)	24.6 (11.8)	3.10 (1.45)	3.40 (1.51)	3.00 (1.33)	3.40 (1.43)	3.20 (1.40)	3.00 (1.41)	2.70 (1.42)	2.70 (1.74)	2.90 (1.37)	3.10 (1.29)	3.40 (1.35)	2.70 (1.64)
Other	290 (6.9)	31.3 (14.9)	2.47 (1.59)	3.26 (1.52)	2.52 (1.56)	2.97 (1.62)	2.84 (1.55)	2.49 (1.49)	2.55 (1.54)	2.42 (2.25)	2.18 (1.46)	3.02 (1.55)	2.82 (1.62)	1.77 (1.32)
Metastatic cancer	299 (7.1)	37.5 (13.6)	3.16 (1.55)	3.75 (1.33)	3.19 (1.44)	3.40 (1.47)	3.44 (1.36)	3.10 (1.50)	3.08 (1.25)	3.03 (1.40)	2.37 (1.40)	3.56 (1.35)	3.40 (1.36)	1.61 (1.20)

## Discussion

Our population-based survey conducted in Shanghai, China, sheds light on the diverse sources and needs for health management information among cancer survivors at various stages of survival. By evaluating the current and anticipated methods of information acquisition among survivors and assessing the information need scores across different cancer types, our findings offer pivotal insights for the future development of patient-centric, long-term follow-up management systems for cancer survivors.

The study reveals that tertiary-level doctors, patient friends, primary care doctors, and secondary hospital nurses constitute the primary sources of information. This preference echoes broader research indicating that Asian patients often favor interpersonal sources of information [29–32]. A systematic review incorporating 112 articles summarized cancer survivors’ information needs and sources, highlighting healthcare professionals (27.3%) as the most common source, with significant needs for information regarding disease stages, treatment options, and side effects [14]. A qualitative study investigating the information needs and preferences of cancer survivors in rural Queensland, Australia, showed that the role of the health professional was critical in providing information and support to rural cancer survivors [33]. Quality information provision after cancer treatment would facilitate improvements in satisfaction among rural cancer survivors. Similarly, Eisfeld et al. studied the information needs and satisfaction among German cancer survivors, identifying support groups, lectures, and oncologists as the most helpful sources [34]. Rutten et al. showed that despite overall increases in cancer information seeking and access to health information from a variety of sources, healthcare providers remain a key source of health information for cancer survivors [35]. The reliance on oncologists reflects the widespread belief in their extensive clinical experience and specialized knowledge, providing professional treatment information and health management strategies [34, 36, 37]. A study investigating the information-seeking behaviors and experiences of cancer survivors in Singapore at various stages of their cancer survival trajectory shows that around half of the searchers (55%) obtained cancer information from the internet [38]. The most preferred source for cancer information was the internet, followed by healthcare professionals [38, 39]. Marchetti et al. investigated health information-seeking behaviors and technology use among skin cancer survivors and found that the internet was most often cited as being the first source that was recently used for health or medical information (45.6%). Compared to skin cancer survivors younger than 65 years old, those 65 years of age or older were more likely to see a doctor first for important health information [40]. The role of patient friends in offering emotional support and facilitating information exchange about the disease, treatments, and side effects is significant. These patient friends can mitigate feelings of fatigue, depression, and, anxiety and enhance the overall quality of life for survivors [41]. Moreover, alternative sources such as the internet and other media present additional opportunities for information support [42]. The internet has become an important medium of health education [40, 43, 44], but behaviors and attitudes are associated with age [45, 46]. Information-seeking is prevalent across all survivorship stages.

This study represents the first instance of documented substantial distinctions in current information sources (tertiary hospital nurses, patient friends) and anticipated sources (secondary hospital nurses) among cancer survivors at various stages. The absence of prior research on this differential makes it challenging to draw definitive conclusions. Statistically significant differences in the sources of information utilized by cancer survivors at different stages of survival, particularly concerning tertiary hospital nurses and patient friends, highlight the evolving information requirements as survivors progress. A possible explanation might be that, with extended survival, patients become more adept at navigating various information sources, while their need for supportive care diminishes. A review investigating cancer patients’ information needs found that preferences and priorities indeed change over time [47]. A study reported that cancer survivors have a significantly lower need for supportive care at the time of treatment and follow-up than at the stage of new diagnosis [19]. Several studies conducted in countries with well-developed healthcare systems have also shown that survivors who have just completed treatment have high rates of unmet needs, while the needs of recovering patients have declined [48]. At the same time, differences in individual information desires should be acknowledged, as not everyone benefits from the same amount of information [49].

Regarding different cancer types, patients with kidney cancer, metastatic cancer, and nasopharyngeal cancer exhibited the highest information need scores. Among primary cancers, breast cancer, colorectal cancer, and tracheal, bronchial, and lung cancers were most common. Breast cancer survivors showed particular interest in carcinogenic factors and medical insurance. For colorectal cancer survivors, high-interest information included carcinogenic factors, medical insurance, and survival rates. Tracheal, bronchial, and lung cancers patients prioritized carcinogenic factor information, followed by medical insurance information. Metastatic cancer survivors expressed a higher need for information on carcinogenic factors, medical insurance, and survival rate. The diagnosis of cancer represents a profound psychological challenge for patients and their families, given the limited curability of most cancers even with early detection, leading to prolonged illness and, in some cases, aggressive and fatal outcomes. Cancer survivor desire information on carcinogenic factors to help slow disease progression, extend survival, improve quality of life, and enhance self-management capabilities [50]. Adequate and appropriate information can increase cancer survivors’ hope and alleviate psychological burdens, enabling better control over their cancer and coping with the uncertainty of the disease [51, 52]. Similar to our findings, a review of 24 studies found that most cancer survivors sought prognosis and survival information [53]. Chien-Wen et al. noted many cancer survivors also desired information on their treatment costs [54]. A study focusing on the unmet informational needs of advanced cancer survivors in China identified economic concerns as a significant issue for both patients and their caregivers, with some families even considering forgoing treatment due to financial constraints [55]. Although oncologists generally fulfill patients’ needs for diagnostic and treatment-related information, they are less likely to provide comprehensive information regarding treatment costs and long-term prognosis. Addressing the need for information related to financial support and medical insurance could not only enhance the confidence of cancer survivors and their families in pursuing treatment but also potentially extend their survival rates.

One of the strengths of our study is the inclusion of a quota sample of cancer survivors from all 16 districts in Shanghai, reducing regional disparities and providing a representative snapshot of the city’s situation. Shanghai, one of China’s most urbanized cities with rich medical resources and excellent disease control, serves as a model for health policy. Our results on the information sources and unmet needs of cancer survivors at different stages can enhance patient satisfaction and care quality, reduce anxiety about cancer and treatment, correct misconceptions and misinformation about cancer, improve adherence to treatment plans and doctor-patient communication, ensure patients’ mental and psychological health, and provide evidence-based support for medical decision-making and health service practices. Nevertheless, our study has limitations. Due to the cross-sectional design, inferences about causality are limited, and we cannot further speculate on the changes in unmet needs of cancer patients over time. Furthermore, the study did not explore potential reasons behind the information sources and needs, such as emotional states and belief systems. Finally, we used a representative regional sample rather than a specific cancer dataset, a choice that enhances the generalizability of our findings but necessitates future research employing specific cancer datasets to validate their sustainability.

## Conclusions

Our study reveals distinct preferences for current and desired sources of information among cancer survivors in China across various stages of survival. Healthcare professionals emerge as the most favored providers of information. The diverse and intricate unmet informational needs across different cancer types underscore the critical role of information in Chinese cancer patients’ efforts to manage their condition. Provision of sufficient information has the potential to facilitate informed decision-making, enhance satisfaction with treatment plans, alleviate psychological barriers, and strengthen confidence and self-management capabilities. Future cohort studies that include larger populations of cancer survivors are needed to provide higher-quality research evidence.

## Data Availability

Due to the protection of the privacy of the study participants, the dataset of this study is not suitable for public disclosure.
